# Congenital anomalies in neurofibromatosis 1: a retrospective register-based total population study

**DOI:** 10.1186/s13023-017-0756-4

**Published:** 2018-01-15

**Authors:** Jussi Leppävirta, Roope A. Kallionpää, Elina Uusitalo, Tero Vahlberg, Minna Pöyhönen, Juha Peltonen, Sirkku Peltonen

**Affiliations:** 1 Department of Dermatology, University of Turku, Turku, Finland; 2 Department of Dermatology, Turku University Hospital, TE6, Hämeentie 11, P O Box 52, FI-20521 Turku, Finland; 3 Institute of Biomedicine, Department of Cell Biology and Anatomy, University of Turku, Kiinamyllynkatu 10, FI-20520 Turku, Finland; 4 Turku University Hospital, Turku, Finland; 5 Department of Biostatistics, University of Turku, Kiinamyllynkatu 10, FI-20520 Turku, Finland; 6 Department of Medical and Clinical Genetics, University of Helsinki, P O Box 160, FI-00029 Helsinki University Hospital, Helsinki, Finland; 7 Department of Clinical Genetics, HUSLAB, Helsinki University Central Hospital, Helsinki, Finland

**Keywords:** Neurofibromatosis type 1, Congenital malformation, Birth defect, Rasopathy, Anomaly, Heart, Kidney, Epidemiology, Face, Polydactyly

## Abstract

**Background:**

Neurofibromatosis type 1 (NF1) is a dominantly inherited Rasopathy caused by mutations in the *NF1* gene on chromosome 17. NF1 has been connected to congenital anomalies, e.g., in the skeletal and cardiovascular systems, but the overall incidence of anomalies is unknown. In this retrospective register-based total population study conducted in Finland, the congenital anomalies in NF1 were evaluated.

**Methods:**

One thousand four hundred ten patients with NF1 were identified by searching the medical records related to inpatient and outpatient hospital visits of patients with an associated diagnosis for NF1 in 1987–2011. Each diagnosis was confirmed by a thorough review of the medical records. Ten non-NF1 control persons per NF1 patient were collected from the Population Register Centre. NF1 patients and controls were linked to the Medical Birth Register and the Register of Congenital Malformations. Odds ratios (OR) and 95% confidence intervals (95% CI) for major congenital anomalies (MCA) were calculated.

**Results:**

The OR for at least one MCA among NF1 children was almost threefold (adjusted OR 2.78, 95% CI 1.71–4.54) compared to controls matched for age, sex and municipality. NF1 children had a significantly increased risk of congenital anomalies in the circulatory (adjusted OR 3.35, 95% CI 1.64–6.83), urinary (adjusted OR 4.26, 95% CI 1.36–13.35) and musculoskeletal (adjusted OR 2.77, 95% CI 1.09–7.02) systems. Also, anomalies of the eye, ear, head and neck were more common among NF1 children than controls (adjusted OR 4.66, 95% CI 1.42–15.31). Non-NF1 children of mothers with NF1 did not have more anomalies than controls (adjusted OR 0.53, 95% CI 0.13–2.21).

**Conclusions:**

Children with NF1 have more MCAs than controls and close follow-up during pregnancy and the neonatal period is required if the mother or father has NF1. Non-NF1 children of mothers with NF1 do not have an increased risk for anomalies.

## Background

Neurofibromatosis 1 (NF1) is a dominantly inherited syndrome that predisposes to cancer. It is caused by mutations in the *NF1* gene on the long arm of chromosome 17 (17q11.2) [1, 2]. The incidence of NF1 is 1:2000–1:3000 and, as the *NF1* gene is prone to mutations, approximately half of the patients present with de novo mutations [3–8]. NF1 is a multisystem disorder affecting all organ systems; the spectrum of symptoms includes osteoporosis [9], learning disability [10], pregnancy and delivery complications [11], cardiovascular abnormalities [12, 13], speech defects [14] and cancer [15]. The diagnosis of NF1 is based on the clinical criteria set by the National Institutes of Health (NIH) and includes café-au-lait macules, neurofibromas, freckling of the flexural areas, optic gliomas, iris hamartomas, distinctive osseous lesions and a first-degree relative with NF1 [16]. If there is a suspicion of NF1 but the clinical criteria are not fulfilled, the diagnosis may be confirmed by mutation analysis.

The *NF1* gene codes for the tumor suppressor protein neurofibromin. It is a very large gene with approximately 280 kb genomic DNA containing 57 constitutive exons and 4 alternatively spliced exons. The gene product neurofibromin is a Ras-GTPase activating protein which inhibits the Ras signaling pathway and interacts with many other proteins [1, 2, 17]. NF1 thus belongs to the group of Ras pathway syndromes, the Rasopathies. Neurofibromin is ubiquitously expressed during embryonic development [18] and it is involved in the differentiation of the skeletal [19], cardiovascular [13, 20] and nervous systems [21]. Hence, it is not surprising that NF1 has been connected to many congenital anomalies, e.g., heart defects [12], vascular anomalies [22] and skeletal anomalies [23]. However, epidemiological studies and large clinical studies on congenital anomalies of patients with NF1 are scarce. Lin et al. (2000) [12] reviewed the cardiologic anomalies of 2322 patients with NF1 in the National Neurofibromatosis Foundation International Database. The overall prevalence of cardiovascular anomalies was 2.3%, which is higher than expected. In particular, the frequency of pulmonic stenosis and aortic coarctation was increased. Ruggieri et al. (1999) [23] studied 135 children with NF1 in the Italian neurofibromatosis clinic, and found that 12 (8.8%) children had congenital bone anomalies. Vertebral and costovertebral anomalies, as well as polydactylies, were more common among children with NF1 than in the general population. Also, other Rasopathies are associated with congenital anomalies, e.g. of the cardiovascular, skeletal, and renal systems [24]. The present study is apparently the first retrospective register-based study on congenital anomalies among patients with NF1 covering the population of a single country, Finland.

## Methods

Patients with NF1 were identified by searching through the electronic medical records of all outpatients and ward patients attending at secondary and tertiary hospitals with a diagnosis of NF1 between January 1987 and December 2011 in mainland Finland. The study population is described in detail by Uusitalo et al. (2015) [4]. Before inclusion into the study cohort, the medical records of each patient were carefully reviewed to confirm that the diagnosis of NF1 fulfilled the NIH clinical criteria [16]. For controls, ten persons per patient with NF1 matched for sex, age and domicile (municipality) were acquired from the Population Register Centre of Finland. First-degree relatives of NF1 patients were censored from the control cohort. For 26 patients with NF1 the full number of control persons was not reached because of the small size of the municipality.

Each resident in Finland has an individual personal identity code, which includes date of birth and gender. As the code remains immutable over the lifetime, it can be used to follow up persons and cross-link data between the national registers. For analyses, the personal identity codes were replaced by randomly generated study person codes to ensure anonymity. The codes of the study persons were also used to form NF1-control sets, each consisting of a person with NF1 and corresponding matched controls. Each set was given an individual group code. The registers covering the time from January 1st 1987 until December 31st 2013 were scrutinized for patients with NF1 and matched controls.

The Register of Congenital Malformations contains data on congenital structural anomalies, chromosomal aberrations and maternal background. The register covers all live births and stillbirths in Finland. In addition, information on induced abortions due to congenital anomalies are collected into the register. The registration of data into this register began in 1963. The data is collected from health care professionals, hospitals, and cytogenetic laboratories, and includes the ICD-9 (International Classification of Diseases 9) codes for the diagnosis and descriptive diagnoses [25]. For the present study, only major congenital anomalies (MCA), as described by the EUROCAT (European surveillance of congenital anomalies) [26], were included in the analysis. Anomalies in the register are entered as ICD-9-codes. Since the ICD-10-classification system is currently used in clinical practice in Finland, the ICD-9-codes were converted to ICD-10-codes manually by reviewing the ICD-9-code and the descriptive diagnosis of each individual anomaly. Anomalies were classified into subgroups according to the classification of congenital anomalies, deformations and chromosomal aberrations in the ICD-10-classification of diseases. Anomalies which are included in the diagnostic criteria of NF1, i.e. sphenoid dysplasia, typical long-bone abnormalities, scoliosis, pseudarthrosis and iris Lisch nodules were excluded from the analysis. Also, cases reported as hamartomas of the brain in the Register of Congenital Malformations were excluded, because they often represent unidentified bright objects (“UBOs”) which are hyperintense regions frequently seen on T2-weighted magnetic resonance brain scans of patients with NF1. Twins were excluded from the analysis of MCAs.

Information in the Register of Congenital Malformations was linked to data in the Medical Birth Register, which contains data on all live births and stillbirths of fetuses with a birth weight of at least 500 g or a gestational age of at least 22 weeks [27, 28]. The Medical Birth Register includes data on maternal background, delivery, pregnancy and the neonate. The data in the register is entered by health care personnel in the hospital of delivery.

The overall incidence of MCAs of the children with NF1 was compared with the children in the matched control group. Subgroup analysis was carried out by stratifying NF1-related cases by the NF1 status of the mother. The incidence of MCAs was also compared between infants of NF1 mothers and of matched controls. Subgroup analysis was also performed by stratifying infants of NF1 mothers by the NF1 status of the infant. Persons with at least one MCA were considered as cases. For the analysis of organ-specific anomalies, the same case could appear in several organ groups if the case had multiple MCAs in different organ groups, but the same case could appear only once in each organ group. In the case of congenital syndromes, which consist of multiple connected anomalies, only the actual syndrome was included as an anomaly (ICD-10: Q80-Q89) and other syndrome-related anomalies were excluded from the analysis. NF1 is not always diagnosed at the time of birth but most cases can be diagnosed by the age of 5 [29], so only children born before 2007 were included in the analysis. The birth size of the newborns was classified according to the International Societies of Pediatric Endocrinology and Growth Hormone Research Society [30]. Finnish birth size curves [31] were used for classification. Small for gestational age (SGA) was defined as birth weight and/or length more than 2 standard deviations (SD) below the gestational age and sex adjusted reference mean. Similarly, large for gestational age (LGA) was defined as a birth weight and/or length more than 2 SDs above the reference mean.

Odds ratios (OR), 95% confidence intervals (CI) and two-tailed *P* values for anomalies were calculated. *P* values <0.05 were considered statistically significant throughout the study. A mixed effects logistic regression was used to calculate adjusted and unadjusted odds ratios for binary variables. A linear mixed model was used to analyze continuous variables. Case-control matching and multiple offspring were taken into account with random intercepts for case-control matching and mother in the mixed models. When the statistical models did not converge with two random intercepts, only random intercept for mother was used, because the variance in the outcomes was higher at the mother level than in the case-control matching level. Analyses of parity were performed by Poisson regression with the person code of the mother as a random variable. The models were adjusted for smoking during the pregnancy, age of mother, year of the pregnancy and parity (0/1+), as they were regarded as clinically relevant confounding factors. The numbers of missing confounding factors among children with NF1 and the matched control children are shown in Table 1. For mothers with NF1 and their matched controls, age of the mother and year of the pregnancy were fully reported and there was no missing data. The smoking status was missing in 18 (5.0%) pregnancies and parity in 2 (0.6%) pregnancies of the mothers with NF1. The numbers in the matched control groups were 114 (2.6%) and 13 (0.3%), respectively. All cases with missing outcome data or confounding variables were excluded from the analysis of the corresponding outcome variable. Statistical analyses were performed with the statistical software SAS version 9.4.

**Table 1:** Baseline characteristics of mothers and offspring

Characteristic	Mothers of offspring with NF1 (*n* = 443)	Mothers of control children (*n* = 4550)	*P*
Age (y)	29.5 ± 5.4	29.0 ± 5.1	.071
Smoking during pregnancy			.300
Yes	73 (16.5)	671 (14.7)	
No	360 (81.3)	3771 (82.9)	
Missing	10 (2.3)	108 (2.4)	
Married or cohabiting			.123
Yes	392 (88.5)	4141 (91.0)	
No	46 (10.4)	381 (8.4)	
Missing	5 (1.1)	28 (0.6)	
Socioeconomic position^a^			
Upper white	40 (11.2)	572 (15.5)	.048
Lower white	151 (42.2)	1603 (43.5)	.857
Blue-collar	84 (23.5)	706 (19.1)	.035
Other	55 (15.4)	589 (16.0)	.874
Missing	28 (7.8)	219 (5.9)	
Parity			.669
1+	278 (62.8)	2742 (60.3)	
0	162 (36.6)	1786 (39.3)	
Missing	3 (0.7)	22 (0.5)	
Sex, offspring			.784
Male	241 (54.4)	2476 (54.4)	
Female	202 (45.6)	2074 (45.6)	
Gestational age, offspring (weeks)	39.2 ± 1.9	39.8 ± 1.6	<.001
Missing	4 (0.9)	29 (0.6)	
Birth size, offspring			
SGA	29 (6.5)	230 (5.1)	.241
AGA	363 (81.9)	4042 (88.8)	<.001
LGA	46 (10.4)	239 (5.3)	<.001
Missing	5 (1.1)	39 (0.9)	

Data are n (%) or mean ± standard deviation. *SGA* small for gestational age, *AGA* appropriate for gestational age, *LGA* large for gestational age

^a^ data available since 1991 (*n* = 358 for mothers of offspring with NF1 and 3689 for mothers of control children)

The study complied with the Declaration of Helsinki and the protocol of the study was approved by the Ethics Committee of the Hospital District of Southwest Finland. Permissions to run the study were obtained from the National Institute for Health and Welfare, and from secondary and tertiary referral centers in Finland.

## Results

The study cohort consisted of 1410 patients (678 males and 732 females). A total of 465 children with NF1, including 22 twins, born before 2007 were identified in the cohort and, for them, 4671 matched controls, including 121 twins. In 119 singleton pregnancies the mother of the neonate with NF1 had also NF1 herself. Among mothers with NF1, 176 females gave birth to a total of 375 children, including 18 twins, during the study period 1987–2013. The corresponding figures in the control group of mothers were 2261, 4511 and 112, respectively. Three pregnancies of the mothers with NF1 were terminated due to congenital anomaly. Among the control mothers 35 pregnancies were terminated due to congenital anomaly.

The baseline characteristics, including the number of missing values, of the children and their mothers are presented in Table 1. The mothers of offspring with NF1 were more often blue-collar workers (OR 1.38, 95% CI 1.06–1.80) and less often upper white-collar workers (OR 0.70, 95% CI 0.49–1.00) than controls but there were otherwise no significant differences between the groups regarding maternal background. The average gestational age of the children with NF1 was 4.2 days (95% CI 2.6–5.6) shorter than of the controls. The children with NF1 were more often large for gestational age than the controls (OR 2.18, 95% CI 1.56–3.06). Table 2 shows the baseline characteristics in relation to MCAs. Small birth size was associated with an increased occurrence of congenital anomalies (OR 2.85, 95% CI 1.60–5.07).

**Table 2:** Baseline characteristics of mothers and offspring in relation to major congenital anomalies

Characteristic	No major congenital anomaly (*n* = 4886)	Major congenital anomaly (*n* = 107)	*P*
Age (y)	29.1 ± 5.2	29.1 ± 5.7	.946
Smoking during pregnancy			.127
Yes	722 (14.8)	22 (20.6)	
No	4047 (82.8)	84 (78.5)	
Missing	117 (2.4)	1 (0.9)	
Married or cohabiting			.255
Yes	4439 (90.9)	94 (87.9)	
No	415 (8.5)	12 (11.2)	
Missing	32 (0.7)	1 (0.9)	
Socioeconomic position^a^			
Upper white	596 (15.1)	16 (16.7)	.771
Lower white	1717 (43.5)	37 (38.5)	.225
Blue-collar	764 (19.3)	26 (27.1)	.098
Other	630 (15.9)	14 (14.6)	.634
Missing	244 (6.2)	3 (3.1)	
Parity			.171
1+	2965 (60.7)	55 (51.4)	
0	1896 (38.8)	52 (48.6)	
Missing	25 (0.5)	0 (0.0)	
Sex, offspring			.993
Male	2659 (54.4)	58 (54.2)	
Female	2227 (45.6)	49 (45.8)	
Gestational age, offspring (weeks)	39.8 ± 1.7	39.4 ± 2.1	.072
Missing	33 (0.7)	0 (0.0)	
Birth size			
SGA	245 (5.0)	14 (13.1)	<.001
AGA	4320 (88.4)	85 (79.4)	.002
LGA	277 (5.7)	8 (7.5)	.521
Missing	44 (0.9)	0 (0.0)	

Data are n (%) or mean ± standard deviation. *SGA* small for gestational age, *AGA* appropriate for gestational age, *LGA* large for gestational age

^a^ data available since 1991 (*n* = 3951 for children with no congenital anomaly and 96 for children with congenital anomaly)

The overall incidences and ORs of congenital anomalies among the children in this cohort, stratified by NF1 status of the child and the mother, are presented in Table 3. The overall incidence of congenital anomalies was significantly higher among children with NF1 than matched controls. The increased risk was significant regardless of the NF1 status of the mother. In contrast, the incidence of MCAs was not increased among non-NF1 children born to mothers with NF1.

**Table 3:** Incidence and odds ratios of major congenital anomalies, stratified by NF1 status of mother and child

Mother / child	*n* ^a^	Incidence, 1/1000	OR, unadjusted (95% CI)	OR, adjusted (95% CI)	*P*, unadjusted	*P*, adjusted
NF1 or non-NF1 / NF1^b^	22	49.7	2.77 (1.70–4.51)	2.78 (1.71–4.54)	<.001	<.001
NF1 / NF1 ^b^	7	56.5	3.44 (1.50–7.88)	3.27 (1.42–7.52)	.004	.005
NF1 / non-NF1 ^b^	2	13.8	0.50 (0.12–2.04)	0.53 (0.13–2.21)	.332	.387
Non-NF1 / NF1 ^b^	15	47.9	2.66 (1.49–4.74)	2.66 (1.48–4.78)	.002	.002
NF1 / NF1 or non-NF1^c^	17	47.6	1.61 (0.93–2.77)	1.65 (0.94–2.89)	.089	.079

Adjusted ORs were adjusted for smoking during pregnancy, maternal age, year of pregnancy and parity (0/1+)

^a^Number of patients with major congenital anomaly

^b^Children born between 1987 and 2006

^c^Children born between 1987 and 2013

The incidences and ORs of anomalies of children with NF1 and matched controls, classified by organ system, are presented in Table 4. There were 22 children with NF1 and 82 controls who had some form of congenital anomaly; of these 2 children with NF1 and 2 controls had congenital anomalies in more than one organ system. Children with NF1 had significantly more anomalies in the circulatory, urinary, and musculoskeletal systems than controls. Also, anomalies of the eye, ear, head, and neck were more common among NF1 children than controls.

**Table 4:** Odds ratios of major congenital anomalies among NF1 children compared to matched controls

Anomaly (ICD-10 code)	NF1 (*n*)	NF1 (1/1000)	Controls (*n*)	Controls (1/1000)	OR, unadjusted (95% CI)	OR, adjusted (95% CI)	*P*, unadjusted	*P*, adjusted
Nervous system (Q00-Q07)	0	0	3	0.7	NA	NA	NA	NA
Eye, ear, face and neck (Q10-Q18)	3	6.8	7	1.5	4.43 (1.12–17.57)	4.66 (1.42–15.31)	.035	.011
Circulatory sytem (Q20-Q28)	9	20.3	25	5.5	3.74 (1.74–8.04)	3.35 (1.64–6.83)	<.001	<.001
Respiratory system (Q30-Q34)	0	0	0	0	NA	NA	NA	NA
Cleft lip and cleft palate (Q35-Q37)	0	0	11	2.4	NA	NA	NA	NA
Other digestive system (Q38-Q45)	1	2.3	4	0.9	2.92 (0.33–25.78)	NA	.335	NA
Genital organs (Q50-Q56)	0	0	1	0.2	NA	NA	NA	NA
Urinary system (Q60-Q64)	4	9.0	11	2.4	4.27 (1.36–13.37)	4.26 (1.36–13.35)	.013	.013
Musculoskeletal system (Q65-Q79)	6	13.5	22	4.8	2.83 (1.12–7.11)	2.77 (1.09–7.02)	.028	.032
Other (Q80-Q89)	1	2.3	3	0.7	3.89 (0.41–36.92)	3.88 (0.41–36.90)	.237	.238
Chromosomal (Q90-Q99)	0	0	0	0	NA	NA	NA	NA

Adjusted ORs were adjusted for smoking during pregnancy, maternal age, year of pregnancy and parity (0/1+)

*NA* Not enough events for statistical analysis

The frequencies of the individual anomalies among children with NF1 are shown in Table 5. The medical records of the children with NF1 with an anomaly of the urinary system were reviewed with special regard to plexiform neurofibromas, potentially explaining the findings in the abdominal or pelvic areas but none were found. Also, the medical records of NF1 patients with anomalies of the eye, ear, head or neck were carefully reviewed. There was one case of congenital glaucoma which turned out to be secondary to a plexiform neurofibroma and was excluded from the analysis. Otherwise, there were no plexiform neurofibromas to explain the congenital anomalies of the head and neck.

**Table 5:** Number of individual congenital anomalies among children with NF1

Congenital anomaly	Number
Eye, ear, face and neck	
Coloboma of iris	1
Ptosis	2
Circulatory system	
Ventricular septal defect	1
Patent ductus arteriosus (gestational age ≥ 37 weeks)	2
Subvalvular aortic stenosis	1
Aortic valve insufficiency	1
Ostium secundum atrial septal defect	1
Pulmonary valve stenosis (gestational age ≥ 37 weeks)	2
Arteriovenous malformation of brain	1
Other digestive system	
Anorectal atresia with fistula	1
Urinary system	
Hydronephrosis (dilatation >10 mm)	3
Double ureter	1
Musculoskeletal system	
Polydactyly	5
Syndactyly	2
Craniosynostosis (middle sagittal suture)	1
Other	
Fetal alcohol syndrome	1

Out of the 465 children with NF1 in our cohort, 129 had diagnosis for NF1 recorded in the Register of Congenital Malformations. Café-au-lait macules were registered for 14 of the children with NF1, neurofibroma for 1, freckles in the flexural regions for 3, optic glioma for 5, iris hamartomas for 2, pseudarthrosis/bowing of the limb for 6, thoracic scoliosis for 1, hamartomas of central nervous system for 3 and plexiform neurofibroma for 1. These NF1 related anomalies were not included in the analysis of the incidence of the congenital anomalies.

## Discussion

This is the first study showing beyond doubt that persons with NF1 have an increased risk for major congenital anomalies but that such anomalies are not more common among the healthy children of mothers with NF1 than among controls.

The risk for MCAs of the circulatory system was significantly increased among persons with NF1, which is consistent with previous studies on cardiac anomalies in this [12, 13, 32], and other Rasopathies [33]. The frequency of pulmonary valve stenosis was high among persons with NF1 in the study by Lin et al. [12]. In the current study, two infants with NF1 had pulmonary valve stenosis which supports prior data that the risk for pulmonary valve stenosis may be increased among persons with NF1. Also, the incidence of musculoskeletal MCAs was increased, as also reported previously by Ruggieri et al. [23] who found an increased frequency of polydactyly among children with NF1. Also in our study, polydactyly occurred among children with NF1.

Our study demonstrated novel findings showing that the anomalies of the urinary system and anomalies in the group of eye, ear, face and neck are more common among children with NF1 than in controls. The medical records of these patients were reviewed to minimize the possibility of plexiform neurofibromas explaining the anomalies. Only one case of anomaly reported in the Register of Congenital Malformations was found to be secondary to plexiform neurofibroma and was not accounted as a case in the analysis, indicating that the true frequency of anomalies in these organ groups is increased. However, the absence of plexiform neurofibromas was not systematically examined with magnetic resonance imaging (MRI). Plexiform neurofibromas are often difficult to diagnose clinically [34], and there is a possibility that these patients have, in fact, plexiform tumors but these tumors have not been diagnosed.

The cohort of patients with NF1 was acquired independently of the Register of Congenital Malformations by examination of the electronic medical records of all outpatients and ward patients attending at secondary and tertiary hospitals with a diagnosis of NF1. This reduces the risk of incorrectly high frequency of anomalies among patients with NF1. However, possible bias cannot be fully eliminated, since persons with anomalies may have more hospital visits which would increase the possibility of being diagnosed with NF1. As congenital anomalies are actively collected into the register from children up to 1 year of age [25], the register must have less information about anomalies diagnosed after this period. Due to relatively small population of Finland (5,451,270 on 31st December 2013) only the overall incidence of congenital anomalies and incidence of anomalies in the selected organ groups can be evaluated reliably. The population size does not allow evaluation of separate anomalies, which could lead to a more precise hypothesis of the mechanisms behind the anomalies among patients with NF1. As often with register-based studies, the study cohort can be biased towards more difficult manifestations of NF1. On the other hand, children with extremely severe manifestation of the disease, such as cerebral infarction, hemorrhage or fatal brain tumor, may never survive long enough to a get the diagnosis of NF1. Males are slightly overrepresented in our group of children with NF1, which may be due to an earlier age at diagnosis of NF1 among boys and an increased mortality of girls before the age of 5. In the future, international collaborative studies are needed to establish if certain anomalies are significantly more frequent in the NF1-population than in controls and to study the long-term morbidity to the patients incurred by these anomalies. This could lead to more detailed guidelines for follow-up and management of NF1-related pregnancies.

## Conclusions

Children with NF1 have more anomalies than controls. Since the increased frequency of congenital anomalies may also reflect an increased risk for severe anomalies, close follow-up during the pregnancy and neonatal period is required if the mother or father has NF1. Close attention should be especially paid to identifying any signs of abnormalities in the cardiovascular or urinary systems and there should be a low threshold for performing imaging studies to find conditions requiring treatment or follow-up. However, approximately half of the children with NF1 are born to the parents without NF1 and follow-up during the pregnancy follows the regular routines. The fact that healthy children of mother with NF1 do not have an increased risk for congenital anomalies is also significant when considering the need of monitoring the infant.

## Abbreviations

AGA: Appropriate for gestational age
CI: Confidence interval
DNA: Deoxyribonucleic acid
EUROCAT: European Surveillance of Congenital Anomalies
GTP: Guanosine triphosphate
ICD: International Classification of Diseases
LGA: Large for gestational age
MCA: Major congenital anomaly
MRI: Magnetic resonance imaging
NF1: Neurofibromatosis type 1
NIH: National Institutes of Health
OR: Odds ratio
SD: Standard deviation
SGA: Small for gestational age
UBO: Unidentified bright object
